# Does low level laser therapy relieve the pain caused by the placement of the orthodontic separators? — A meta-analysis

**DOI:** 10.1186/s13005-015-0085-6

**Published:** 2015-08-28

**Authors:** Quan Shi, Shuo Yang, Fangfang Jia, Juan Xu

**Affiliations:** Department of Stomatology, Chinese People’s Liberation Army General Hospital, 28 Fuxing Road, 100853 Beijing, China

**Keywords:** Pain, Orthodontic separators, Low level laser therapy, Analgesic effect, Meta-analysis

## Abstract

**Objective:**

Pain caused by orthodontic treatment can affect patient’s compliance and even force them to terminate treatments. The aim of this meta-analysis is to evaluate of the analgesic effect of low level laser therapy (LLLT) after placement of the orthodontic separators.

**Methods:**

Five databases: PubMed, Embase, Cochrane library, China Biology Medicine disc (SinoMed CBM), China National Knowledge Infrastructure (CNKI) were searched for all the appropriate studies in June, 2014. Two reviewers screened the research results under our inclusion criteria and evaluated the risk of bias independently. Then the data of the included studies was extracted for quantitative analysis by the Review Manager 5.1 software.

**Results:**

Six studies were included in our meta-analysis finally. Comparing to the placebo group, the LLLT has good analgesic effect at 6 h, 1d, 2d, 3d after placement of separators which is of statistical significance. While at 2 h, 4d, 5d after the placement, the results tend to support LLLT, but not statistically significant.

**Conclusion:**

Based on current included studies, LLLT can reduce the pain caused by the placement of separators effectively. However, because of the high heterogeneity, well designed RCTs are required in the future.

## Introduction

Pain is a subjective experience and a common clinical symptom in orthodontic patients. Research shows that as many as 95 % of orthodontic patients will feel pain and 8-30 % of patients discontinue treatment because of pain [1–3]. Sometimes pain can affect patient’s compliance and therefore affect treatment effect. Despite the orthodontic technology has been great developed, the issue of pain has not been solved very well.

Many orthodontic operations can cause pain [2, 4–7]. As a common and necessary operation, placement of separators to create enough space for bands can cause mild to moderate pain [8]. It is generally believed that when periodontal ligament under pressure, the mediators of inflammation are released, such as prostaglandins, histamine, substance P ,which cause sensitivity to the free nerve terminations and pain or discomfort after placement of archwires or separators [2, 9]. In several methods currently available, the medication is thought to be the most effective [10], especially the non-steroidal anti-inflammatory drugs (NSAIDs). Some articles [1, 9–11] proved that they can relieve orthodontic pain effectively. But the medication also has its side effects which cannot be ignored: allergy and inhibiting tooth movement [10, 12]. Therefore, the application of medication is limited.

There are no effective clinically proven non-invasive, non-pharmacological methods used to relieve the pain caused by orthodontic. But some studies showed that low level laser maybe have analgesic effect [5, 13–20]. Low level laser, or low level laser therapy(LLLT), is a new internationally accepted designation and defined as laser treatment in which the energy output is low enough so as not to cause a rise in the temperature of the treated tissue above 36.5 °C or normal body temperature[20]. LLLT can inhibit the development of inflammation [21, 22], accelerate of bone repair [23], increase the rate of teeth movement [24]. Besides, LLLT have been used to treat temporal-mandibular joint disorder [25], relive the pain after teeth extraction [26].

As a non-invasive method, with no report of serious adverse effect events [10], LLLT is better than drugs in clinical application prospect. But there is still a lack of reliable evidence to prove that LLLT can effectively reduce the orthodontic pain. So the aim of this systematic review is to collect the randomized controlled trials (RCTs) or controlled clinical trials (CCTs) about LLLT relive the pain of patients after placement of separators and evaluate of the analgesic effect of LLLT.

## Material and methods

The methods for this review were based on the Cochrane Handbook for Systematic Reviews of Interventions [27]. In the whole process, the studies were assessed by 2 observers independently and any disagreement will resolved by discussion. The data was analyzed by the Review Manager 5.1 software.

### Literature search and study selection

The following electronic databases were searched in June 2014 without time and language restricted: PubMed, Embase, Cochrane library, China Biology Medicine disc (SinoMed CBM), China National Knowledge Infrastructure (CNKI). The search strategies of PubMed, Embase and Cochrane library were showed in Table 1.

**Table 1:** Search strategy and results for T pubmed, Embase and cochranme library

Database	Search strategy	Result
pubmed	#1: pain OR discomfort OR toothache	591803
	#2 :(low power laser) OR ( low level laser ) OR LLLT OR (low output laser) OR (low intensity laser)	10881
	#3: orthodontic*	52498
	#4 : #1 AND #2 AND #3	33
EMBASE	#1: pain OR discomfort OR toothache	1054689
	#2 : (low power laser) OR ( low level laser ) OR LLLT OR (low output laser) OR (low intensity laser)	19478
	#3: orthodontic*	61408
	#4: #1 AND #2 AND #3	49
The Cochrane library	#1: (pain OR discomfort OR toothache) AND laser AND orthodontic*	42

### Inclusion criteria

The following selection criteria were applied.

1. Design: the studies should be designed as RCT or controlled clinical trial (CCT), including split-mouth design.
2. Participants: patients received elastomeric separators on the premolar or molar.
3. Interventions and comparators: low level laser therapy (LLLT) vs placebo. (For some studies, there are not only these two groups, if we can filter out the data we need from the studies, we will include them either.)
4. Outcome: measurement of the pain after placing the elastomeric separators.

### Exclusion criteria

The exclusion criteria were as follows:

1. In vitro study (laboratory studies and animal studies), case report or letters.
2. Study without available data can not be used by our meta-analysis.
3. The pain was caused by other operations of orthodontic instead of placing the elastomeric separators.
4. The participants had systemic disease or chronic pain or histories of neurologic and psychiatric disorders and other characteristics which will have influence on the outcome.

### Data extraction

We designed a table to collect the experimental information and data which include the author, country, year of publication, design type, number of participant, measure method, the pain value and standard deviation, and so on. Then use a new table to record the parameters of the laser and the treatment regimen.

### Risk of bias evaluation

Totally seven items need to be taken into consideration: (1) allocation concealment, (2) random sequence generation,(3)blinding of participants and personnel, (4) blinding of outcome assessment, (5) incomplete outcome data, (6) selective reporting, (7) other bias. The risk of bias for each item was judged as low risk, high risk, or unclear risk. The overall risk of bias for the each study was evaluated by the following criteria:

If the risk of bias is low for all the items, the study is of low risk.

If one (or more than one) of the risk of bias is high for the key items, the study is of high risk.

If one (or more than one) of the risk of bias is unclear, the study is of unclear risk.

### Data analysis

The meta-analysis was performed by combining the results of the included studies which had measured the pain at the same evaluation intervals for the continuous data. In addition, chi^2^ and I^2^ was used to estimate the degree of heterogeneity. Mean differences, standard deviations, and 95 % confidence intervals (CI) were to be calculated for individual trials and overall effect using a random effects model or a fixed effects model for continuous data.

## Results

### Searching and selection results

**Fig. 1:**
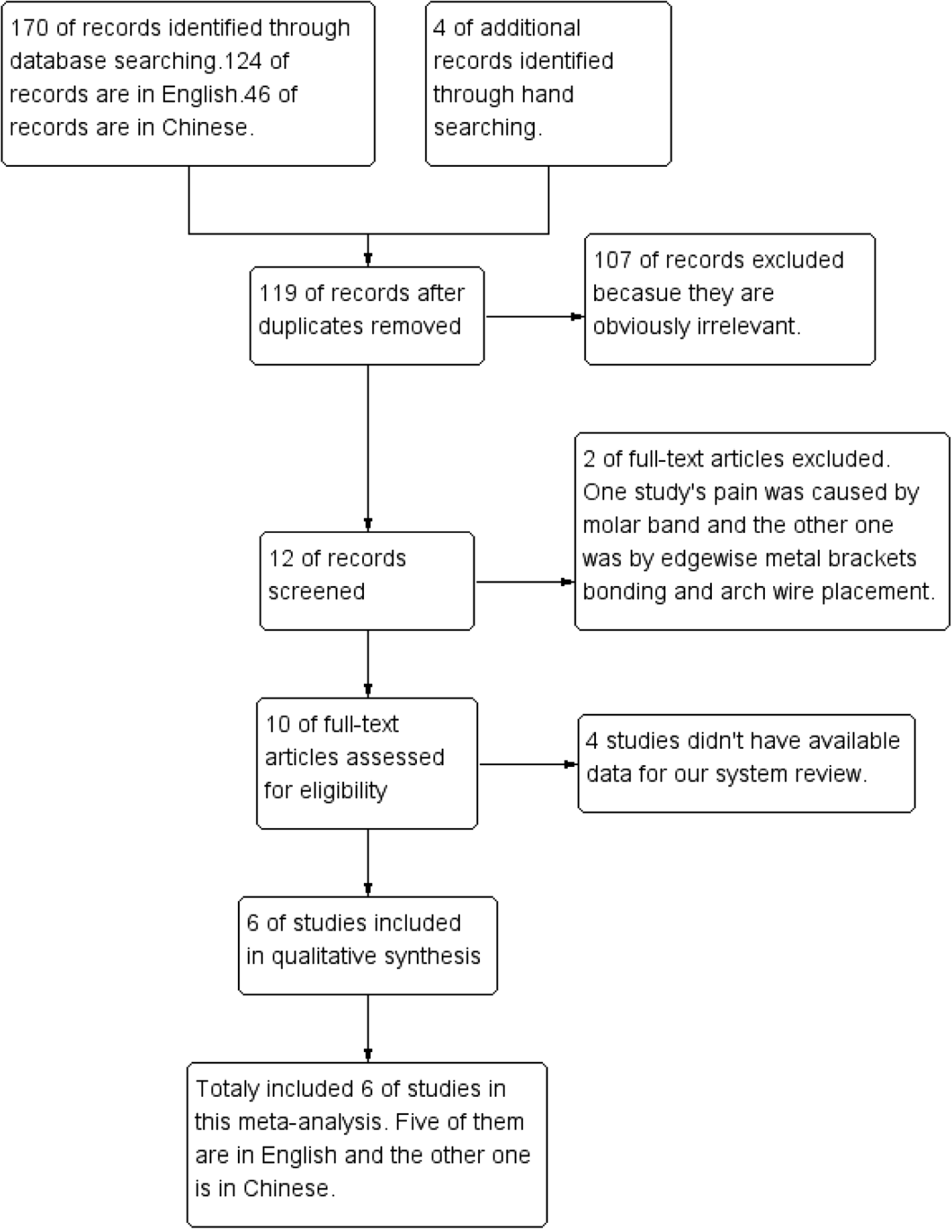
Study flow diagram

The selection progress is shown in Fig. 1. After reading the full-text of the 10 potential interests [13–17, 19, 20, 28–30], we found that five articles [13–15, 20, 29] have available data for our meta-analysis. For the rest studies, we contacted the authors of the articles by sending e-mail (except Lim HM et al. 1995 because there is no e-mail address in the article). But only one author [19] sent us the data we needed. Finally, we include six studies [13–15, 19, 20, 29] in our meta-analysis. Five of them [13–15, 19, 29] are in English and the other one [20] is in Chinese.

### Characteristics of the included studies

The detailed descriptions of the characteristics about the six included studies are shown in Tables 2, 3 and Table 4. In the six studies we included, five of them are RCT [13, 14, 19, 20, 29], and one is CCT [15]. Six studies encompassing 295 subjects. One study [15] used a split mouth design method. Five studies [13–15, 20, 29] placed the separator on the mesial and distal of the first molar, and one [19] placed separator on the first, second premolar and the first molar at the same time (totally four separators per subjects).

**Table 2:** Characteristics of included studies

Study ID	Country	Design	Number (P/L)^a^	Average age	Separators
Celestino No´brega 2013	Brazil	RCT	60 (30/30)	17.5	3 M Unitek
Won Tae Kim 2012	Korea	RCT	58 (30/28)	21.52	Dentalastics Separators, Dentaurum,Ispringen, Germany,2.1 mm
Ladan Eslamian 2013	Iran	CCT (split mouth design)	37 (37/37)	24.97	Dentarum, Springen, Germany
Esper MA 2011	Brazil	RCT	38 (38/12)	23.4	Morelli, 4.0 mm, Ø 5/32"
Ida Marini 2013	Italy	RCT	80 (40/40)	23.0875	NR^b^
Zhang HY 2014	China	RCT	60 (30/30)	15.9	NR

^a^:P = placebo group; L = LLLT group

^b^:NR = not report

**Table 3:** Characteristics of included studies

Study ID	Teeth	Intervention method	Evaluation intervals	Pain measure method
Celestino No´brega 2013	mesial and distal sides of the first permanent lower molars on the left and right sides	each subject received irradiation one spot on the region of root apex, three points along the root axis on the buccal side	2 h,6 h,24 h,3 d,5 d	VAS ;The incidence of free of pain
Won Tae Kim 2012	mesially and distally on both of the maxillary first molars.	apply laser for 30 seconds on each area immediately then every 12 hours for 1 week with close contact between the tip and mucosa to irradiate the mesiobuccal, mesiolingual, distobuccal, and distolingual areas.	5 min,1 h,6 h,12 h,1 d,2 d,3 d,4 d,5 d,6 d,7 d	VAS
Ladan Eslamian 2013	first permanent molars (distal and mesial),either on maxillary (22 patients) or mandibular (15 patients) arches	laser irradiation on the buccal side (at the cervical third of the roots), for distal and mesial of the second premolars and first permanent molars, as well as distal of second permanent molars (five doses) . The same procedure was repeated for the lingual or palatal side (five doses). After 24 h, patients returned to the clinic and received another 10 doses of laser irradiation on the same quadrant.	0 h,6 h,24 h,30 h,3 d,4 d,5 d,6 d,7 d	VAS
Esper MA 2011	Placebo :mesial and distal of the first upper and lower molar on the right side while the Laser group on left side	Radiation was applied punctually, touching the gum perpendicularly on two points of the vestibular side and on the lingual side of the separated molars, both points were in the cervical and radicular region	pre-placement 2 h,24 h,48 h,72 h,96 h	VAS
Ida Marini 2013	right first ,second premolar and first molar (upper arch or lower arch)	The laser probe was applied on the cervical third of buccal and lingual gingiva l covering of each root.	0 h,12 h,24 h,36 h,48 h,72 h,96 h	VAS,Questionnaire
Zhang HY 2014	First molar	the laser probe was 5 mm away from the mucosal ,Laser irradiation was applied on first molar root apical ,then Move up along the long axis of the tooth to the tooth neck (totally 4 points)	2 h,6 h,24 h,72 h,120 h	VAS

**Table 4:** Detail of the lasers and parameters

Study ID	Laser type	Wave length (nm)	Output power (mW)	Number of irradiated points or area (cm2)	Irradiation time	Frequency	Dose (J/cm2)	Field diameter
Celestino No´brega 2013	aluminum gallium arsenide diode laser	830	40.6	4 points	25 s per each 1 J/cm2, totally 125 s	after placing the separator	root apex 2 J/cm2,the other three points was 1 J/cm2, totally 5 J/cm2	2 mm
Won Tae Kim 2012	semiconductor laser device with an AlGaInP diode	635	6	4	30 seconds on each area	every 12 h for 1 week	NR^a^	5.6 mm
Ladan Eslamian 2013	Ga-Al-As laser	810	100	10	20 s	laser was applied immediately and 24 hours later after placing the separators	2	NR
Esper MA 2011	InGaAlP laser	660	30	4	25 s each point	after placing the separator	4 J/cm2 per point, totally 16 J/cm2 per tooth	5 mm
Ida Marini 2013	GaAs diode laser superpulsed wave	910	160	6	totally 340 s	The irradiation started immediately after placing orthodontic separators.	NR	8 mm
Zhang HY 2014	semiconductor laser	650 and 830	30	4	30S each point,totally 120 s per tooth	after placing the separator	NR	3-5 mm

^a^NR = not report

The detail of the lasers and parameters are shown in Table 4. The wavelength of the laser ranged from 635 nm to 910 nm. One study [20] used a mix of 650 nm and 830 nm. All the studies used a semiconductor laser. The output power ranged from 6 mW to 160 mW.

All the included studies used VAS to evaluate the pain. The mean pain values and standard deviations of laser group and placebo group at each evaluation interval of the six studies are collected. In one study [19], the data was got from the author by sending e-mail. Although all of the studies used the VAS score to evaluate the pain, but the score ranged from 0 to 100 in two studies [14, 19]and the other four studies [13, 15, 20, 29] ranged from 0 to 10. However, all of them use the same method to evaluate the pain in each group. Therefore, the data of these two studies were converted to centesimal system.

### Risk of bias evaluation

**Fig. 2:**
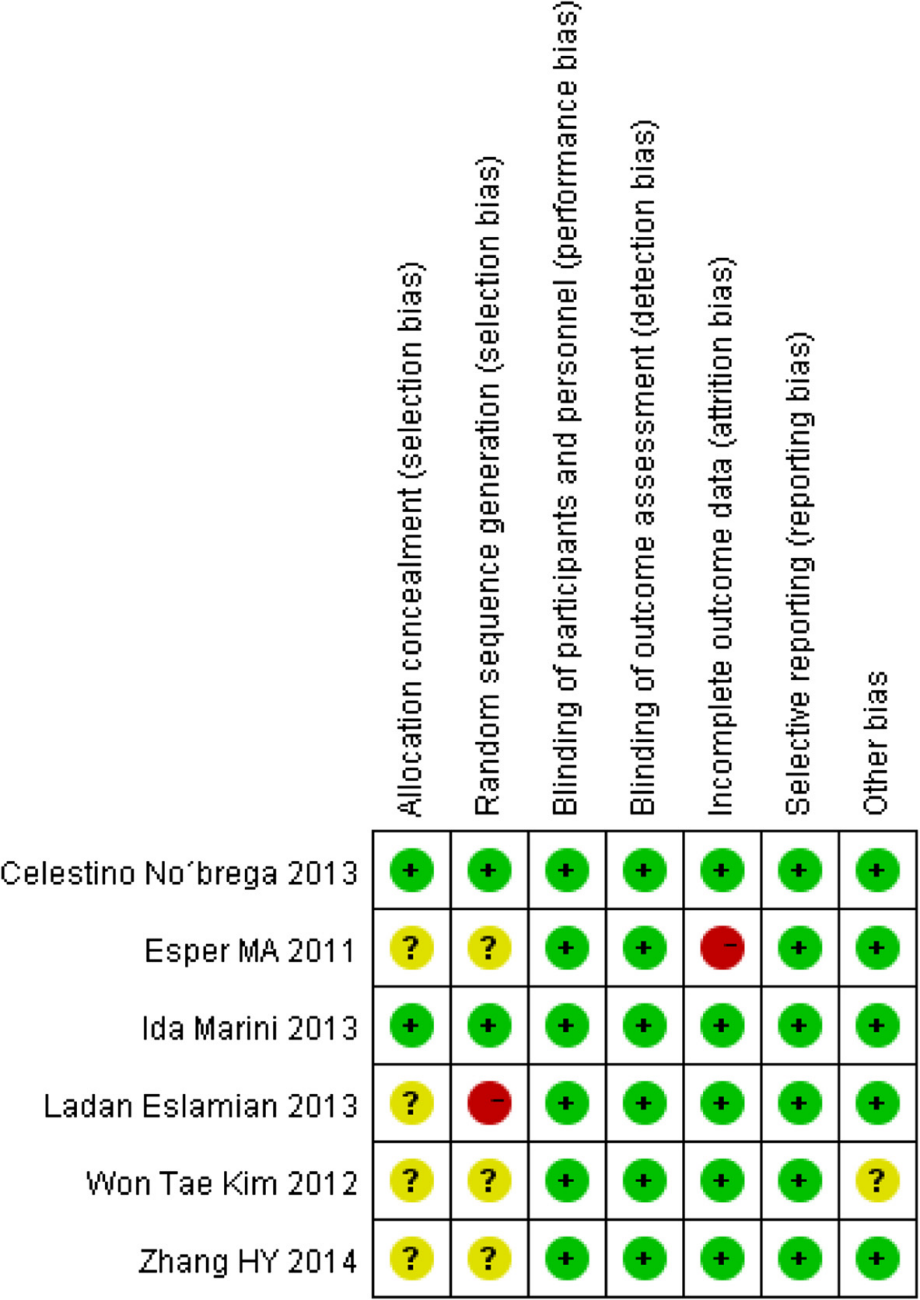
Risk of bias for every study. Of the six included studies, two [13, 19] of them were judged to have a low risk of bias because all the items were of low risk of bias and one study [19] is a random, triple-blinding, placebo control clinic trail while the other one [13] is a random double-blinding, placebo control clinic trail. Two [14, 20] of the six studies were judged to have an unclear risk of bias, because the authors failed to describe the method of randomization and had no report of the allocation concealment. At the same time, the study of Won Tae Kim, et al. [14] was judged to have unclear bias on the item of “other bias” because the application of the laser was performed by the subjects at home, so there may be compliance bias. Two studies [15, 29] were judged to have a high risk of bias because one of the studies [15] used inappropriate method of randomization and there was a subject drop out without details description in the study of Esper MA, et al. [29]

The risk of bias summary is shown in the Fig. 2. If there is inadequate information in the article, we will contact the author by e-mails or seek advice from statisticians. Of the six included studies, two [13, 19] of them were judged to have a low risk of bias. Two studies [14, 20] were judged to have an unclear risk of bias. Two studies [15, 29] were judged to have a high risk of bias .

### Meta-analysis for mean score of pain

**Fig. 3:**
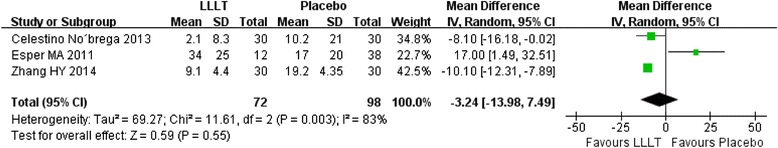
Forest plot of pooled mean difference at 2 hours

**Fig. 4:**
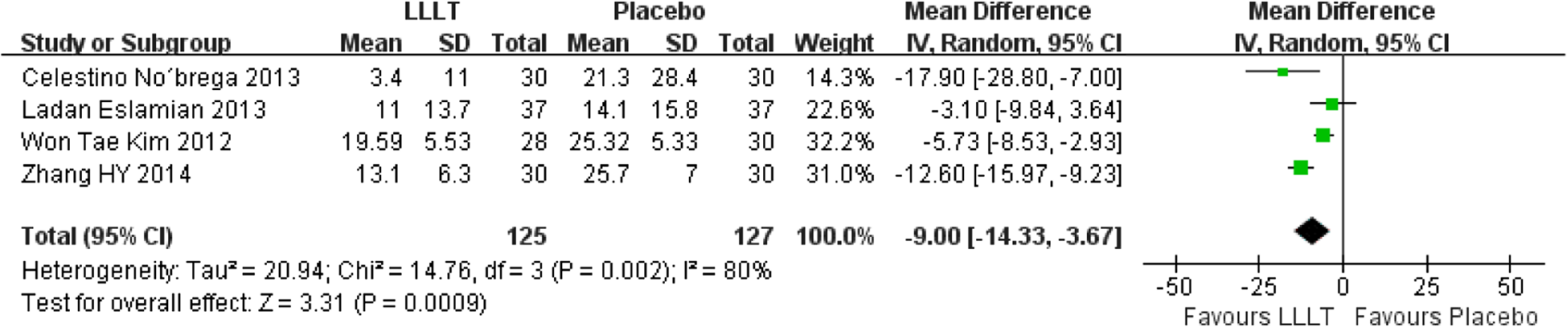
Forest plot of pooled mean difference at 6 hours

**Fig. 5:**
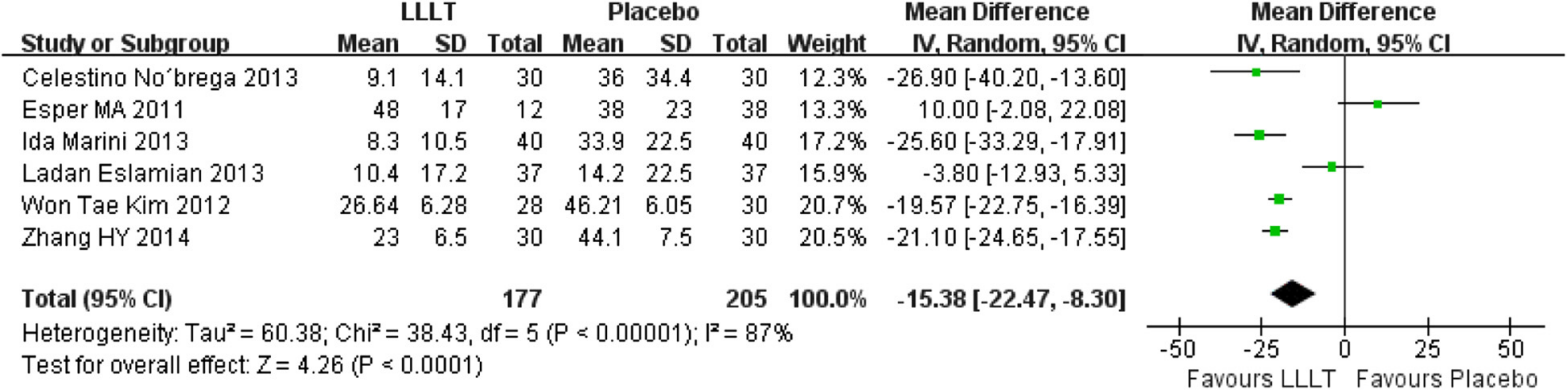
Forest plot of pooled mean difference at 1 day

**Fig. 6:**
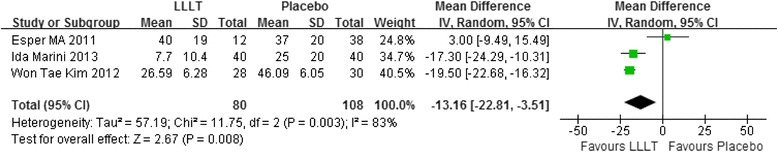
Forest plot of pooled mean difference at 2 day

**Fig. 7:**
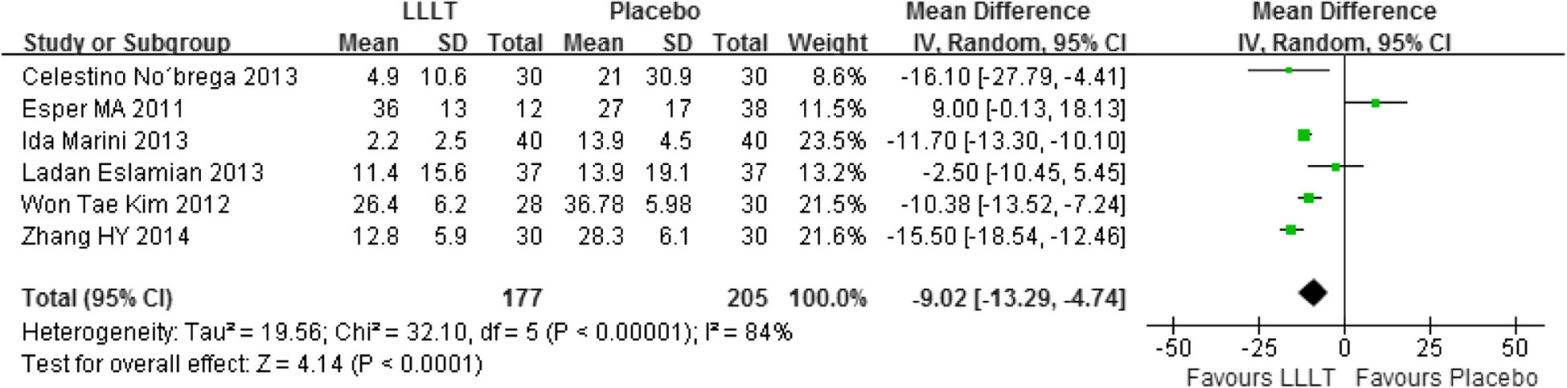
Forest plot of pooled mean difference at 3 day

**Fig. 8:**
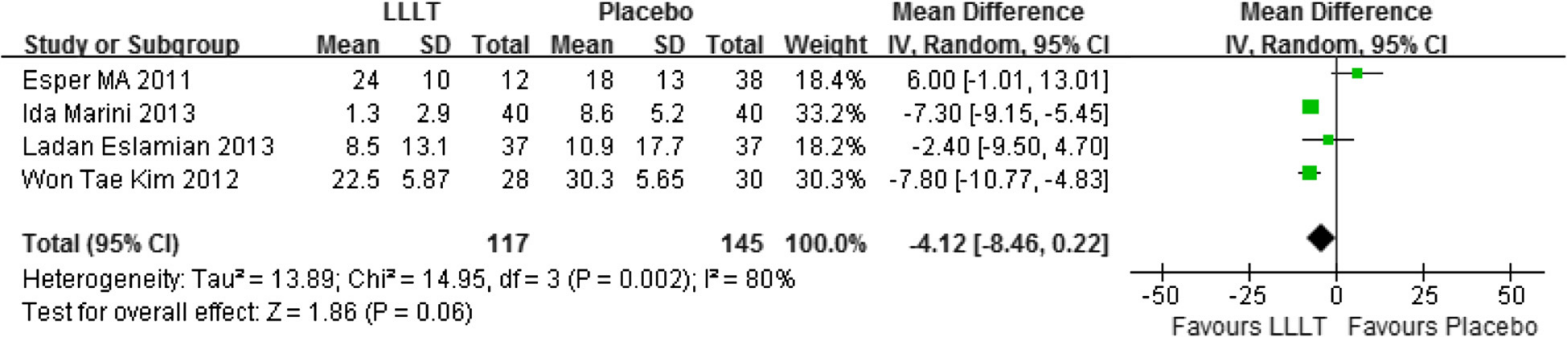
Forest plot of pooled mean difference at 4 day

**Fig. 9:**
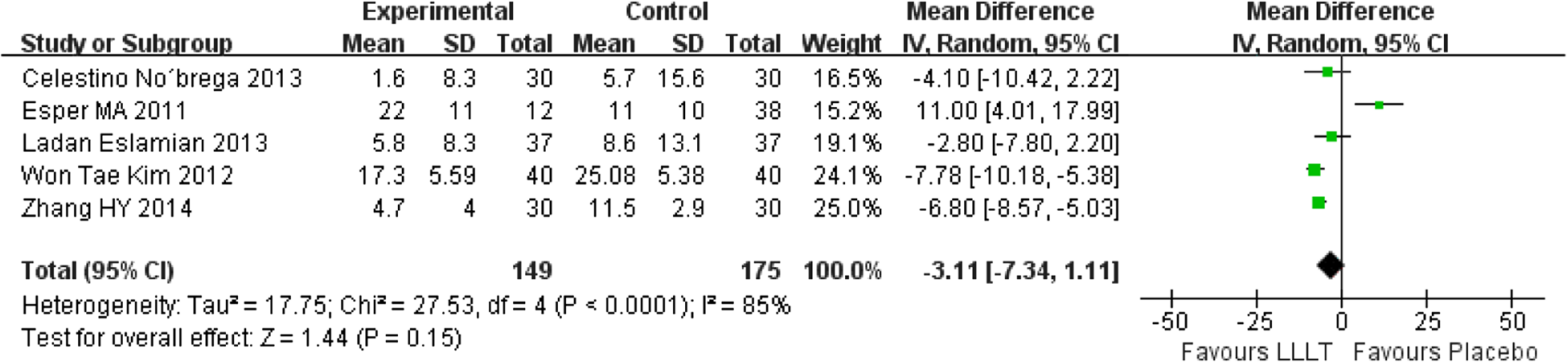
Forest plot of pooled mean difference at 5 day

In our included studies, if there were three or more studies measured the pain score at the same time point, we will make an analysis. Therefore, totally seven time points meet the requirements: 2 hours, 6 hours, 24 hours, 2 days, 3 days, 4 days, 5 days. Figs. 3, 4, 5, 6, 7, 8 and 9 showed the comparison between LLLT and Placebo on pain relief after placing the separators at each time point. Because of the high heterogeneity, a random effect was selected.

2 hours after the placement, the overall effect test showed no significant different between the LLLT and placebo (P = 0.55). The mean difference was −3.24 and 95 % CI( −13.98 , 7.49) (Fig. 3).

While for the time points of 6 h, 24 h, 2d, 3d, the overall effects favored the LLLT and showed a statistical difference between the LLLT and placebo, because all of the P values of the tests were less than 0.05 (Figs 4, 5, 6 and 7).

At 4^th^ day and 5^th^ day the overall effects showed there was no statistical difference between the LLLT and the placebo group (P = 0.06 at 4d and P = 0.15 at 5d).

### The pain incidence

One of the included studies reported the rate of pain never appeared and never disappeared [19]. The result showed that 30 % of the LLLT group subjects did not feel pain while the placebo group was 0 %. In another study [13], the proportion of subjects reporting the absence of pain was significantly higher in LLLT group at each time point. Meta-analysis is not feasible because of inadequate data.

## Discussion

Pain caused by orthodontic treatment can affect patient’s compliance and change their eating habits [8], even forcing them to terminate treatments [13]. Orthodontists have been working on the controlling of pain. Although the NSAIDs had been proved effective on pain control, the side effects limited its clinical application [9–12]. Some researches [5, 13–20] consider LLLT as an effective method to control orthodontic pain, therefore this system review is to confirm this analgesic effect after placement of separators. Because many orthodontic operations can induce pain, in order to reduce the heterogeneity of clinical, we select the studies of using LLLT to relief pain after placing the separators.

For the orthodontic treatment with fixed appliances, the separators were used to create enough space for the bands[8]. After placement, whether separators or arch wires, the periodontal ligament and the vessels were under pressure, causing the release of inflammatory mediators and inducing pain [2, 9].

However, it is difficult to measure the pain precisely because pain is a subjective experience, the individual variability of pain threshold and sensitivity can be influenced by physical and psychological effects [18, 19]. Besides, other factors, such as environmental, sociocultural, genetic factors, and so on, can influence pain [15]. Therefore, from these viewpoints, the split-mouth design perhaps is the best choice. In our included studies, only one is split-mouth design. There are no objective measurements for pain. The VAS is one of the most common used tools to measure pain intensity at present [8, 16]. All of the six included studies in this review used this method. What’s more, in order to avoid the psychological effect, we need well designed clinical trials to evaluate the pain. Using placebo is one of our included criteria, which would increase the reliability of the results and decrease the psychological effects. Two of the six included studies used red light [19] or light-emitting diode(LED) [14] whose intensity was very low compared to the laser. The other four studies used pseudo-laser as placebo. Only two studies [13, 19] reported the correct random sequence generation method and allocation concealment.

In our meta-analysis, compare to the placebo group, the LLLT has good analgesic effect and the results favored the LLLT at 6 h, 1d, 2d, 3d after placement of separators which is of statistical significance. While at 2 h, 4d, 5d, the results tend to support LLLT without statistically significant. A system review [26] concluded that LLLT modulates biochemical inflammatory markers and produces local anti-inflammatory effects in cells and soft tissue which contribute to relief acute pain in the short-term. Besides, the review found there were strong evidences that LLLT can improve angiogenesis. Because of high heterogeneity of different studies which may be caused by different races, laser parameters, using methods and frequency, bias risk, we chose a random effect model. At present, the most commonly used non-surgical lasers are diode, with a wave length ranging from 600 to 1,000 nm, and potencies between 10 and 100 mW [29]. The wave length of laser used in the six included studies ranged from 635-910 nm and the output power between 6 and 160 mW. All the LLLT in the six studies used semiconductor laser. Besides, the frequency and use method were different in each study. According to some research [5, 13, 15], the laser does not inhibit the cell activity if the dose less than 20 J/cm^2^.The laser doses of included studies were all less than 20 J/cm^2^. At the same time, there were no adverse effects reported by these studies using the lasers under the current parameter ranges.

Two studies [13, 19] report the rate of free of pain (VAS = 0). One [19] report the rate of pain never appeared and the result showed that 30 % of the LLLT group subjects did not feel pain while the placebo group was 0 %. In the other one study [13], the proportion of subjects reporting the absence of pain was significantly higher in LLLT group at each time point. Although it is impossible to make a meta-analysis because of clinical heterogeneity and insufficient data, their results support the effective analgesic effect of LLLT.

According to the results of our meta-analysis, LLLT can reduce the pain caused by the placement of separators effectively without adverse effect under current evidence. Considering LLLT may increase the speed of tooth movement [22], in the field of orthodontics, LLLT may have broad application prospects. But different studies used different separators, different lasers and parameters, different method and frequency of laser, different test positions (mandible or maxilla or both), different design and different risk of bias, and these can lead to the high heterogeneity. Therefore, well designed RCTs are required to evaluate the analgesic effect of LLLT.

## Conclusion

Under current studies and evidences, the results of our meta-analysis reveals that LLLT can reduce the pain caused by the placement of separators effectively at 6 h, 1d, 2d, 3d after the placement of the orthodontic separators without adverse effect reports. Besides, there is no evidence reveals that LLLT can bring forward the most painful day. These results indicate the good clinical application prospect. However, because of the high heterogeneity and the bias risk of included studies, well designed RCTs are required in the future.
